# Association Between Diabetes and Immunoglobulin M Antibodies Against Endogenous Gonadotropin-Releasing Hormone in Serum: A Meta-Analysis

**DOI:** 10.7759/cureus.31415

**Published:** 2022-11-12

**Authors:** Amine Rakab, Karam R Motawea, Samah S. Rozan, Hagar Mahmoud Hamouda, Haidar Alibrahim, Nesreen Elsayed Talat, Rowan Elhalag, Bisher Sawaf, Gihan Mohamed, Adel Albozom, Sarya Swed, Hesham Mohamed Abuelsaoud, Rehab Mohamed Elshazly, Wael Hafez

**Affiliations:** 1 Department of Medical Education, Weill Cornell Medicine Hospital, Doha, QAT; 2 Faculty of Medicine, Alexandria University, Alexandria, EGY; 3 Faculty of Medicine, Aleppo University, Aleppo, SYR; 4 Department of Internal Medicine, Hamad Medical Corporation Hospital, Doha, QAT; 5 Department of Internal Medicine, National Medical Commission Royal Hospital, Abu Dhabi, ARE; 6 Faculty of Medicine, Ain Shams University, Cairo, EGY; 7 Department of Internal Medicine, The National Research Centre, Cairo, EGY

**Keywords:** meta-analysis, gnrh igm antibodies, endogenous gonadotropin-releasing hormone, igm antibodies, diabetes mellitus

## Abstract

Our aim was to perform a meta-analysis to evaluate the possible link between diabetes and high levels of immunoglobulin M (IgM) antibodies against Gonadotropin-Releasing Hormone (GnRH). The search included PubMed, Web of Science, and Scopus databases. Inclusion criteria were any controlled clinical trials or observational studies that measured the level of IgM antibodies against GnRH hormone in diabetic patients, we excluded case reports, editorials, and animal studies. RevMan software, version 5.4 (The Cochrane Collaboration 2020) was used to perform the meta-analysis.

Following the screening, three studies were included in the meta-analysis. The meta-analysis included 99 patients in the diabetes group and 318 healthy persons in the control group. The pooled effect showed no statistically significant association between diabetes and the prevalence of GnRH IgM antibodies compared with the control group (risk ratio {RR} = 1.64, 95% CI = 0.96 to 2.79, p-value = 0.03). The pooled effect showed a statistically significant association between diabetes and increased levels of GnRH IgM antibodies compared with the control group (mean difference {MD} = 2.13, 95% CI = 0.25 to 4.02, p-value = 0.03).

Our study found a significant association between diabetes and increased levels of GnRH IgM antibodies. Therefore, GnRH IgM antibodies may play a role in the pathogenesis of diabetes or may be considered a unique immunological reaction in diabetic patients. More multicenter randomized studies are needed to support our results confirming the positive relationship between diabetes and high levels of IgM antibodies against GnRH hormone.

## Introduction and background

Diabetes mellitus (DM) is a general term for heterogeneous metabolic diseases characterized mainly by chronic hyperglycemia resulting from impaired action of insulin or impaired secretion of insulin or both [1]. DM is one of the world's greatest epidemics affecting both developed and developing countries [2]. According to the most recent estimate by the International Diabetes Federation (IDF), there were 415 million persons with diabetes mellitus in 2015 and the number will reach up to 642 million by 2040 [3]. Diabetes-related chronic hyperglycemia is linked to long-term damage, dysfunction, and failure of multiple organs including the eyes, kidneys, nerves, heart, and blood vessels [4].

Gastrointestinal dysmotility is a common complication of DM which is highly prevalent in the population [5,6]. Gonadotropin-releasing hormone (GnRH) antibodies were detected in the serum of patients with dysmotility and/or DM [7,8]. Gonadotropin-releasing hormone (GnRH) is secreted by the hypothalamus and has a major effect on the pituitary gland as it leads to increasing gonadotropin synthesis and secretion. GnRH is considered a crucial hormone for sexual behavior and reproductive physiology [9]. Peripherally, GnRH and its receptors have been found in the rats’ myenteric plexus and intestinal epithelium. It has also been found in the myenteric neurons of humans [10,11].

A recent study reported that blood glucose levels were significantly affected by intestinal GnRH mRNA levels, indicating that GnRH may be important for glucose homeostasis [12]. Furthermore, long-term GnRH therapy has been associated with the development of diabetes mellitus and autoimmune thyroiditis [13], but other studies reported no association. So, due to these conflicts, we aimed to conduct a meta-analysis to evaluate whether there is an actual link between diabetes and high levels of GnRH IgM antibodies.

This article was previously presented as a meeting abstract at the 19th world congress of insulin resistance & diabetes and cardiovascular diseases conference held from 2-4 December 2021 in the USA.

## Review

Methods

This meta-analysis was performed according to Preferred Reporting Items for Systematic Reviews and Meta-Analyses (PRISMA) guidelines and the Cochrane handbook [14]. We searched PubMed, Web of Science, and Scopus. The following key terms were used; (("GnRH antibodies") OR ("GnRH")) AND ("diabetes mellitus"). Furthermore, the reference list of studies included in this meta-analysis was reviewed to include other relevant studies. The studies were eligible for inclusion if they met the following criteria.

Eligibility Criteria

Any observational studies or randomized clinical trials that reported the level of GnRH IgM antibodies in diabetic patients with no age restriction. We excluded case reports, editorials, conference abstracts, and animal studies. The main outcomes were; levels of GnRH IgM antibodies and the prevalence of GnRH IgM antibodies in diabetic patients.

Screening, Data Extraction, and Risk of Bias

Two reviewers (HA and SS) conducted initial title and abstract screening and discussed all the conflicts to reach an agreement, otherwise, a third opinion from KRM was obtained. Potentially eligible articles were imported for full-text screening and assessed for inclusion. Data was extracted using an Excel sheet. Examples of data collected are study arms, the number of patients in each group, age, sex (n), other baseline diseases, and baseline treatment. The Newcastle Ottawa Scale (NOS) tool was used to assess the included observational studies' quality. Each study was ranked as good, fair, or poor quality.

Data Analysis

Review Manager (RevMan) Software version 5.4 (The Cochrane Collaboration 2020) was used to perform the meta-analysis. The continuous outcomes were measured as mean difference (MD) and standard deviation (SD), and the dichotomous outcomes as risk ratios (RR) with their 95% confidence interval (CI). If heterogeneity was detected (Chi-square P value < 0.05), a random effect model was used otherwise, we used a fixed-effect model, in general; the results were considered significant if the P-value was less than 0.05.

Results

Literature Search

**Figure 1:**
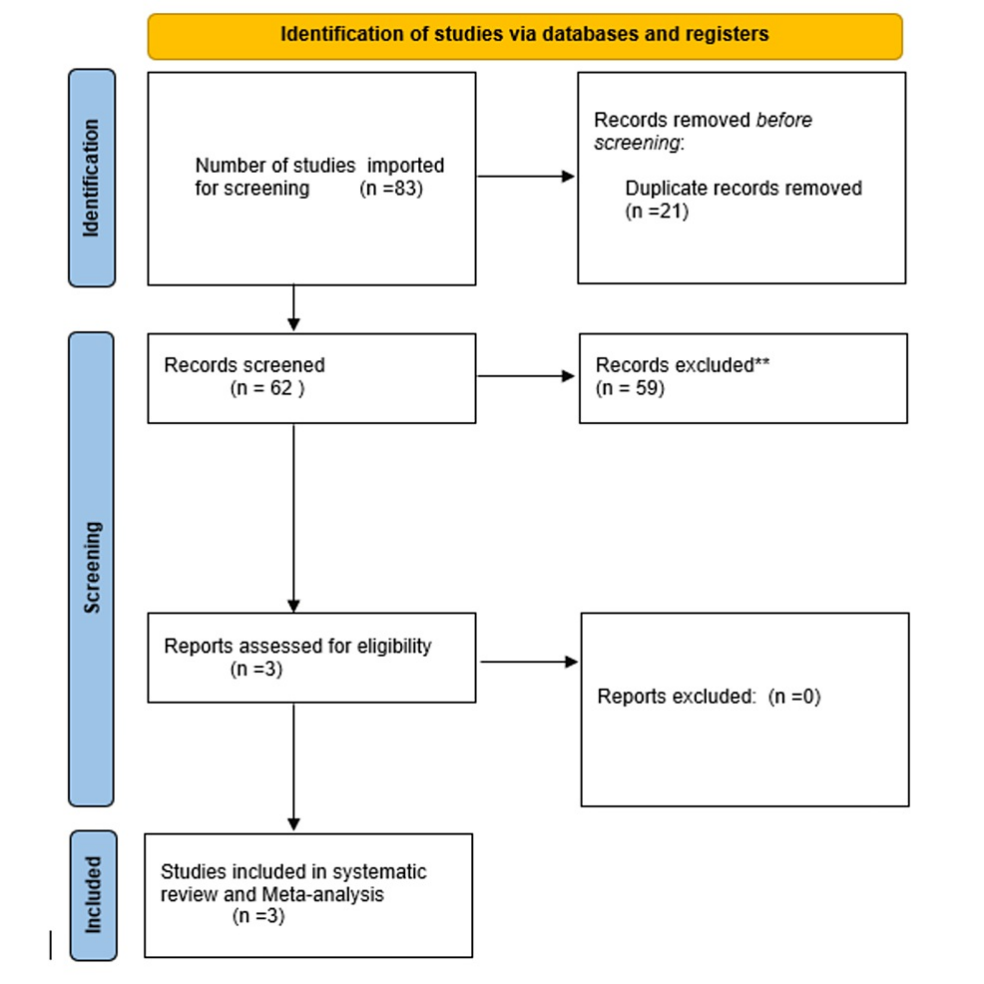
PRISMA flow diagram PRISMA: Preferred Reporting Items for Systematic Reviews and Meta-Analyses Asterisks denote irrelevant studies excluded after the title and abstract screening.

After a comprehensive search of the literature, 83 studies were obtained. Out of those 83 studies, only 62 studies were suitable for the title and abstract screening after the removal of duplicates. Of the 62 studies, 59 were irrelevant and three studies were eligible for full-text screening. Finally, three observational studies were included in the meta-analysis after full-text screening [7,8,15], as shown in the PRISMA flowchart (Figure 1). The summary of the included studies is shown in Table 1.

**Table 1:** Summary of the included studies IgG: Immunoglobulin G, RU: relative units, IBS: Irritable bowel syndrome, IBD: Inflammatory bowel disease, GI: gastrointestinal, GnRH: gonadotropin-releasing hormone, OR: odds ratio, CI: confidence interval, P: p-value, LH: luteinizing hormone, ELISA: enzyme-linked immunosorbent assay

ID	Study design	Country of the study	Study arms	Endpoints	Conclusion
Berntorp et al. (2013) [7]	Cross-sectional study	Sweden	Case: Diabetes patients Control: Healthy Blood donors	Thirty-nine consecutive patients with diabetes mellitus were included in the study after clinical examination and examination by esophageal manometry and gastric emptying scintigraphy. The serum was analyzed for the presence of antibodies against GnRH using an ELISA, and values are expressed as relative units (RU). Two age- and gender-matched healthy subjects per each patient served as controls. The prevalence of IgM GnRH antibodies in patients was 33% compared to 14% in controls (p = 0.027), with a higher antibody titer; 1.2 (0.6-5.0) and 0.2 (0.1-0.3) RU, respectively (p = 0.000). The expression of IgG antibodies was 15% in patients and none in controls (p = 0.000). Lower body mass index was associated with the presence of IgM antibodies (OR = 0.835, 95% CI = 0.699-0.998), and autonomic neuropathy with the presence of IgG antibodies (OR = 9.000, 95% CI = 1.327-61.025). Esophageal dysmotility (69%) or gastroparesis (18%) were not associated with the presence of IgM antibodies (OR = 0.589, 95% CI = 0.143-2.424, and OR = 3.407, 95% CI = 0.633-18.350, respectively). Neither was esophageal dysmotility associated with IgG antibodies (OR = 2.500, 95% CI = 0.259-24.096).	The study concluded that Antibodies against GnRH are more common in patients with diabetes mellitus compared with healthy controls. IgM antibodies are associated with lower body mass index and IgG antibodies are associated with autonomic neuropathy.
Ohlsson, et al. (2011) [8]	Prospective observational study	Sweden	Case: Diabetes patients Control: Healthy Blood donors	Healthy controls expressed low levels of GnRH IgM antibodies with a prevalence of 23%. The prevalence of GnRH IgM antibodies in IBS and dysmotility patients was 42% (P = 0.008), and the levels were higher (P = 0.000). Patients with diabetes mellitus expressed GnRH IgM antibodies in the same prevalence as controls (25%), but at higher levels (P = 0.02). Patients with celiac disease or IBD had the same or lower levels of antibodies. There were no associations between antibodies, other co-existing diseases, or laboratory analyses.	The study concluded that Higher levels of GnRH IgM antibodies were detected in patients with IBS and dysmotility, but not organic GI diseases, compared with healthy controls. These findings suggest that IBS and dysmotility to some extent may be of an autoimmune origin.
Roth et al. (2014) [15]	Prospective observational study	Sweden	Case: Diabetes patients Control: Healthy Blood donors	Patients with gastrointestinal complaints related to diabetes mellitus only expressed IgM antibodies against GnRH1, the prevalence of which tended to be significantly elevated compared with controls (Table 2). Consecutive patients with diabetes mellitus had a higher prevalence of IgM antibodies against progonadoliberin-2 than controls, whereas the expression of IgM and IgG antibodies against GnRH1 was equal (p = 1.000 and p = 1.000, respectively). Except for a tendency toward a shorter half-time of gastric emptying rate in patients with antibodies against progonadoliberin-2 (27.0[26.0–50.5] and 52.5 [36.2–71.438], respectively, p = 0.056), no other clinical associations could be found. None of the patients with diabetes mellitus showed antibodies against the GnRH receptor, LH, or LH receptor. The ELISA for the analysis of GnRH2 was unstable, and for this reason, these data are not shown. The distribution of antibodies was equal in men and women among controls and patients with diabetes mellitus. In patients with IBS and dysmotility, all antibodies were expressed in women.	In conclusion, The study confirms previous results that IgM antibodies against GnRH1 are elevated in serum in patients with IBS, dysmotility, and/or diabetes mellitus compared to controls. Furthermore, also IgM antibodies against progonadoliberin-2 and GnRH receptors are elevated in these patients, whereas IgG antibodies against these peptides, or antibodies against LH or LH receptors are not present. It remains to explain the mechanisms behind the antibody formation and to examine whether gastrointestinal complaints, autonomic neuropathy, or psychological factors are associated with the formation.

The total number of patients included in the study is 417, 99 patients in the diabetes group and 318 patients in the control group, other baseline data are shown in Table 2.

**Table 2:** Baseline characteristics of the included studies IBS: Irritable bowel syndrome, IBD: Inflammatory bowel disease, CIPO: Chronic intestinal pseudo-obstruction, ED: Erectile dysfunction

ID		Study arms		Number of patients in each group		Age (Years)		sex (n)				Duration of diabetes		other baseline diseases	
		Cases	Control	Cases	Control	Cases	Control	Cases		Control					
								female	male	female	Male	Cases	Control	Cases	Control
Berntorp et al (2013) [7]		Diabetes patients	Healthy blood donors served	39	456	52.4		27(69)	12(31)	216	240	26.3	Healthy blood donors	Esophageal dysmotility (27) Gastric dysmotility (7) Retinopathy (28) Angiopathy (13) Microalbuminuria (4) Macroalbuminuria (3) Autonomic neuropathy (10) Peripheral neuropathy (19)	Healthy blood donors
B OHLSSON et al (2011) [8]		Diabetes patients	Healthy blood donors served	20	456	51		11	9	216	240		Healthy blood donors	IBS and dysmotility (40) IBD (34) Celiac disease (96)	Healthy blood donors
Roth et al (2014) [15]		Diabetes patients	healthy blood donors	40	200	51	42	27	13	100	100	31	Healthy blood donors	IBS/dysmotility (n = 45) ED (n = 12) CIPO (n = 5) Gastroparesis (n = 3)	Healthy blood donors

GnRH IgM antibodies prevalence and levels outcomes were compared between the diabetes group and the control group. The overall quality was good in one study and fair in two studies as shown in Table 3.

**Table 3:** Quality assessment of the included studies NOS: Newcastle Ottawa Scale, AHRQ: Agency for Health Research and Quality

NOS scale Risk of Bias Assessment										
	Selection				Comparability	Exposure			Total Score	AHRQ standards
Study	case definition	Representativeness	Selection of controls	Definition of controls	Comparability	Ascertainment	Same method	Non-response rate		
Berntorp et al. (2013) [7]	0	1	1	1	1	1	0	1	6	Fair Quality
Roth et al. (2014) [15]	1	1	1	1	1	1	1	1	8	Good Quality
Ohlsson et al. (2011) [8]	0	1	1	1	1	1	1	1	7	Fair Quality

Outcomes

Levels of GnRH IgM Antibodies

**Figure 2:**
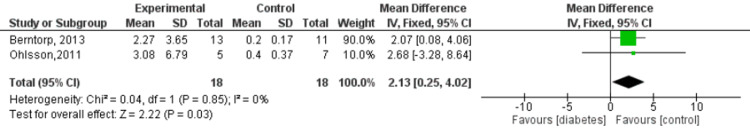
Forest plot of GnRH IgM antibodies outcome References [7,8]

The pooled analysis showed a statistically significant association between diabetes and increased levels of GnRH IgM antibodies compared with controls (MD = 2.13, 95% CI = 0.25 to 4.02, p-value = 0.03). We observed no heterogeneity among studies (p = 0.85, I² = 0%), Figure 2.

Prevalence of GnRH IgM Antibodies

**Figure 3:**
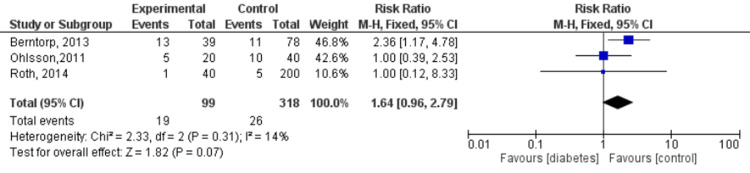
Forest plot of GnRH IgM antibodies prevalence outcome References [7,8,15]

The pooled analysis showed no statistically significant difference between the diabetes and the control group (RR = 1.64, 95% CI = 0.96 to 2.79, p-value = 0.03). We observed no heterogeneity among studies (p = 0.31, I² = 14%), Figure 3.

Discussion

Our meta-analysis showed a statistically significant association between diabetes mellitus and increased levels of IgM antibodies against GnRH compared with the control group but no difference in the incidence of these antibodies was detected. Two studies showed that IgM antibodies against GnRH1 tended to be higher in patients with gastrointestinal complaints related to diabetes mellitus, compared to controls [11,15]. Another study showed lower BMI in diabetes mellitus was associated with the expression of IgM antibodies [7].

In previous studies, it was shown that multiple treatments with GnRH agonists have been associated with enteric dysmotility and antibodies against GnRH, but the patients included in our analysis were not treated with these drugs. The antibodies are assumed to be secondary to dysmotility, reflecting gastrointestinal (GI) neuronal damage [16]. 

GnRH is a crucial regulating hormone in reproductive and sexual behavior [9]. GnRH and GnRH receptors have been found peripherally in the rats’ myenteric plexus and intestinal epithelium [10,17]. In humans, they were only found in the myenteric plexus [11]. A recent report suggested that GnRH may be important for glucose homeostasis by showing a significant effect of intestinal GnRH mRNA level on the blood glucose level [12].

Implications of the study

Diabetic patients should be followed up and serological tests should be done for them regularly. The results of our study give the green light for more research investigating the possible role of GnRH antibodies in the pathogenesis of diabetes opening the way for more diabetes therapeutics targeting GnRH antibodies. The strength of our study is that our analysis showed No heterogeneity observed among studies in the prevalence and the levels of GnRH IgM antibodies outcomes (p = 0.45, I² = 0%), (p = 0.85, I² = 0%), respectively. The exact mechanism of these antibodies' formation and their association with DM are still unclear. Future studies are needed to study this association.

Limitation

Our study is limited by small sample sizes pooled from the present studies. Future observational studies are needed to be done to allow hypothesize the association between DM and the presence of IgM antibodies. However, causality is still difficult to be assessed even with these studies. 

## Conclusions

Diabetes is significantly associated with increased levels of GnRH IgM antibodies compared with the healthy controls. Therefore, GnRH IgM antibodies may play a role in the pathogenesis of diabetes or may be considered as a unique immunological reaction in diabetic patients. Future clinical trials targeting this immunological reaction may play an important role in the management of diabetes. More multi-center randomized studies with larger sample sizes are needed to support our findings and to establish the significant association between diabetes and increased levels of GnRH IgM antibodies.

## Appendices

Authors' contributions: AR and KRM contributed to conceptualization, methodology, formal analysis, writing, reviewing, and editing the original draft; SSS and NET contributed to methodology and formal analysis; HMH, HA, RE, BS, GM, AA, SS, HMA, RNE contributed to data extraction, quality assessment, writing, and reviewing the article; WH contributed to reviewing and proofreading the article.
